# Dr. Baillie

**Published:** 1823-11

**Authors:** 


### OBITUARY.

### DR. BAILLIE.

In our last Number, we promised a Biographical Sketch of this distinguished physician: we could not hope to redeem this pledge so perfectly by any composition of our own, as by the following account of Dr. Baillie, given by Mr. Bell, in his Introductory Lecture; which he has had the politeness to send us for insertion. Mr. Bell had been previously speaking of the Hunters, and, having mentioned Dr. Baillie, he continued—

"Gentlemen,—I have been led unavoidably to mention that name. But I shall not venture to give myself up to the feelings which, at this moment, it could not fail to excite. Indeed, the reflections which arise on the contemplation of a loss so recent and so great, would carry me beyond the terms of praise with which you are as yet prepared to sympathise.

You, who are just entering on your professional studies, cannot be aware of the importance of one man to the character of a profession, the members of which extend over the civilised world. You cannot yet estimate the thousand chances there are against a man rising to the degree of eminence which Dr. Baillie attained; nor know how slender the hope of seeing his place supplied in our day.

The father of Dr. Baillie was the Rev. James Baillie, sometime minister of the kirk of Shotts, (one of the most barren and wild parts of the low country of Scotland,) and afterwards Professor of Divinity in the University of Glasgow. His mother was the sister of Dr. William Hunter and of Mr. John Hunter.

In the earlier part of his education, he enjoyed great advantages; and, indeed, he was in the whole course of it peculiarly happy. From the College of Glasgow, he went to Baliol College, Oxford, where he took his degrees; and came finally under the superintendance of his uncle, Dr. William Hunter. By him he was brought forward into life; and, through his influence, was made physician to St. George's Hospital.

The merest chance brought me acquainted with a circumstance very honourable to Dr. Baillie. While still a young man, and not affluent, his uncle William, dying, left him the small family estate of Longcalderwood. We all know of the unhappy misunderstanding that existed between Dr. Hunter and his brother John. Dr. Baillie felt that he owed this bequest to the partiality of his uncle, and made it over to John Hunter. The latter long refused; but, in the end, the family-estate remained the property of the brother, and not of the nephew, of Dr. Hunter.

It was Dr. Hunter's wish to see his nephew succeed him, and take his place in these rooms as a lecturer. To effect this, he united with him his assistant, Mr. Cruickshanks; and, at his death, assigned to him the use of his collection of anatomical preparations during thirty years.

It was under this roof that Dr. Baillie formed himself, and here the profession learned to appreciate him.

He had no desire to get rid of the national peculiarities of language; or, if he had, he did not perfectly succeed. Not only did the language of his native land linger on his tongue, but its recollections clung to his heart; and to the last, amidst the splendour of his professional life, and the seductions of a court, he took a hearty interest in the happiness and the eminence of his original country. And may the world forget him who forgets this first demand on his gratitude, and best excitement to honourable exertions! But there was a native sense and strength of mind which distinguished him, and more than compensated for the want of the polish and purity of English pronunciation.

He possessed the valuable talent of making an abstruse and difficult subject plain: his prelections were remarkable for that lucid order and clearness of expression which proceed from a perfect conception of the subject; and he never permitted any vanity of display to turn him from his great object of conveying information in the simplest and most intelligible way, and so as to be most useful to the pupils.

It is to be regretted that his association in the lectureship made his duties here unpleasant to him; and I have his own authority for saying that, but for this, he would have continued to lecture some years longer. That Dr. Baillie ceased to lecture at a time when his opinions became every day more valuable, is the less to be regretted when we consider how he continued afterwards to occupy himself.

His first work, on Morbid Anatomy, was, like every thing he did, modest and unpretending; but it was not on that account the less valued. A perfect knowledge of his subject, acquired in the midst of the fullest opportunities, enabled him to compress into a small volume more accurate and more useful information than will be found in the works of Bonetus, Morgagni, and Lieutaud. This work consisted at first of a plain statement of facts,—the description of the appearances presented on dissection, or what could be preserved and exhibited; and he afterwards added the narration of symptoms corresponding with the morbid appearances. This was an attempt of greater difficulty, which will require the experience of successive lives to perfect.

His next work was the Illustration of Morbid Anatomy, by a series of splendid engravings; creditable at once to his own taste and liberality, and to the state of the arts in this country. He thus laid a solid foundation for pathology, and did for his profession what no physician had done before his time. Much, no doubt, remains unperformed; but I am confident that nothing which he has done will be undone by those who shall follow him.

Besides his great work, he gave a description of the Gravid Uterus, and contributions to the Transactions and medical collections of his time.

Dr. Baillie presented his collection of morbid specimens to the College of Physicians, with a sum of money to be expended in keeping them in order; and it is rather remarkable that Dr. Hunter, and his brother and his nephew, should have left to their country such noble memorials as these. In the College of Glasgow may be seen the princely collection of Dr. Hunter; the College of Surgeons have assumed new dignity, surrounded by the collection of Mr. Hunter,—more like the successive works of many men enjoying royal patronage or national support, than the work of a private surgeon; and, lastly, Dr. Baillie has given to the College of Physicians, at least, that foundation for a museum of morbid anatomy, which we hope to see completed by the activity of the members of that body.*

We cannot estimate too highly the influence of Dr. Baillie's character on the profession to which he belonged. I ought not, perhaps, to mention his mild virtues and domestic charities; yet the recollection of these must give a deeper tone to our regret, and will be interwoven with his public character, embellishing what seemed to want no addition. These private virtues ensured for him a solid and unenvied reputation. All wished to imitate his life,—none to detract from his fame. Every young physician, who hoped for success, sought his counsel; and I have heard him forcibly represent the necessity of a blameless life, and that, unless medical reputation be joined with purity of private character, it neither could be great nor lasting.

The same warmth of feeling and generosity which prompted him to many acts of private charity and benevolence, were not without a

* At a meeting of the Royal College of Physicians, on the 30th September, the following tribute of respect to the memory of the late Dr. Baillie, was ordered to be inserted in the College Annals:

"That our posterity may know the extent of its obligations to the benefactor whose death we deplore, be it recorded, that Dr. Baillie gave the whole of his most valuable collection of anatomical preparations to the College, and six hundred pounds for the preservation of the same: and this, too, (after the example of the illustrious Harvey,) in his life-time. His contemporaries need not an enumeration of his many virtues to account for their respectful attachment to him whilst he lived, or to justify the profound grief which they feel at his death. But to the rising generation of physicians it may be useful to hold up, for an example, his remarkable simplicity of heart, his strict and clear integrity, his generosity, and that religious principle by which his conduct seemed always to be governed, as well calculated to secure to them the respect and good-will of their colleagues and the profession at large, and the high estimation and confidence of the public."

powerful influence upon his conduct on more arduous occasions, and may well be supposed to have guided and sustained him in circumstances which might have shaken other men, of less firm and independent minds. But I shall not dwell on this view of his public character. The matters to which I allude are ill fitted for discussion in this place: they belong rather to the history of the period in which he lived, and will there be most suitably recorded.

When you add to what I have said of the celebrity of the uncles, William and John Hunter, the example of Dr. Baillie, and further consider the eminence of his sister, Joanna Baillie, excelled by none of her sex in any age, you must conclude, with me, that the family has exhibited a singular extent and variety of talent.

Dr. Baillie's age was not great, if measured by length of years: he had not completed his sixty-third year; but his life was long in usefulness. He lived long enough to complete the model of a professional life. In the studies of youth,—in the serious and manly occupations of the middle period of life,—in the upright, humane, and honourable conduct of a physician,—and, above all, in that dignified conduct which became a man mature in years and honours, he has left a finished example to his profession.

After so many years spent in the cultivation of the most severe science, —for surely anatomy and pathology may be so considered,—and in the performance of professional duties on the largest scale,—for he was consulted not only by those who personally knew him, but by individuals of all nations,—he had, of late years, betaken himself to other studies, as a pastime and recreation. He attended more to the general progress of science. He took particular pleasure in mineralogy; and, even from the natural history of the articles of the Pharmacopœia, he appears to have derived a new source of gratification.

By a certain difficulty which he contrived to place in the way of those who wished to consult him, and by seeing them only in company with other medical attendants, he procured for himself, in the latter part of his life, that leisure which his health required, and which suited the maturity of his reputation; while he intentionally left the field of practice open to new aspirants.

When I last saw him, (the day before he left town for Tunbridge,) I enjoyed a long and interesting conversation with him. He was aware of his condition and his danger. His friends believed that he was suffering from a general decay of strength,—a sort of climacteric disease. To me he appeared like a man who had some local source of irritation, or visceral affection, which was preying on his constitution.

Every body hoped that his state of health was to be ascribed to the fatigue of business, and that retirement would afford him relief; but in this we were disappointed. He sensibly and rapidly sunk, and, by the calmness and resignation of his last days, summed up the virtues of his life."

---

To the above account we subjoin some further particulars, with reference to the dates.

Matthew Baillie, M.D. was born in the Manse of Tholy, near Hamilton, in October 1761. His father was the Rev. Dr. James Baillie, clergyman of the Presbyterian church; his mother was Dorothea, sister of Dr. and Mr. John Hunter.

He went to the University of Glasgow in his thirteenth year. In 1779, he was appointed to an exhibition at Baliol College, upon which he went to reside at Oxford. In 1780, he went to London, and took up his abode in Dr. Hunter's house, (keeping his terms at Oxford.) In 1785, began to give regular lectures on Anatomy. He lectured on anatomy, in conjunction with Mr. Cruickshank, until 1799. During this period he made a collection of anatomical preparations, which he afterwards presented to the College of Physicians. In the year 1787, he was elected physician to St. George's Hospital: he held this office thirteen years; became doctor of physic in 1789, and immediately afterwards a fellow of the College of Physicians. The work on Morbid Anatomy was published, we believe, in 1795: this went through several editions. The engravings to illustrate the Morbid Anatomy were published in fasciculi, which appeared at intervals; they began to appear in 1799. He was elected fellow of the Royal Society in 1789; he was also a fellow of the Royal Society of Edinburgh, and honorary member of the College of Physicians in Edinburgh.

Dr. Baillie had two sisters, who survive him: one of them, Miss Joanna Baillie, the authoress of Plays on the Passions; and he has left two children, a son and a daughter. Mrs. Baillie was the daughter of Dr. Denman, and sister of the Common Serjeant and Lady Croft.

---

