# Erratum

**Published:** 1823-12

**Authors:** 


ERRATUM.—P. 437, for *variety* of display, read *vanity* of display.

